# Evaluation of inflammatory serum parameters as a diagnostic tool in patients with endometriosis: a case-control study

**DOI:** 10.1038/s41598-025-05719-1

**Published:** 2025-06-20

**Authors:** Mariz Kasoha, Panagiotis Sklavounos, Istvan Molnar, Meletios P. Nigdelis, Bashar Haj Hamoud, Erich-Franz Solomayer, Gilbert Georg Klamminger

**Affiliations:** 1 Department of Gynecology and Obstetrics, Saarland University Medical Center (UKS), Homburg, Germany; 2 Department of Obstetrics and Gynecology, University Medical Center of the Johannes Gutenberg University Mainz, Langenbeckstraße 1, 55131 Mainz, Germany

**Keywords:** Diagnostic markers, Diagnostic markers

## Abstract

Even though non-invasive prediction of endometriosis may seem technically feasible using sophisticated machine learning algorithms, a standard clinical use case for non-surgical diagnosis of endometriosis has not yet been established. In the present paper, we assess the potential of the inflammatory serum markers hepcidin, soluble urokinase-type plasminogen activator receptor (suPar), and interleukin-6 (IL-6) in a cohort of 87 patients. Hereby, 59 patients were histologically diagnosed with endometriosis, whereas other 28 patients served as our non-endometriosis control group. An initial exploratory univariate statistical analysis (Mann-Whitney test) revealed the diagnostic potential of different serum levels of suPar (*p* = 0.024) and IL-6 (*p* < 0.001) between both groups; the formation of a distinct training data set (*n* = 77) subsequently allowed to train a supervised machine learning analysis (tree classifier) employing serum levels of suPar, hepcidin, and IL-6 as predictor variables. Based on an internal 5-fold cross validation, the classifier performance was initially assessed using standard metrics such as sensitivity, positive predictive value, and AUROC curve. Additionally, the algorithm was tested on an external validation (holdout) data set (*n* = 10), showing sufficient overall accuracy of 80% without tendencies of overfitting. In conclusion, our data demonstrates the diagnostic potential of IL-6 and suPar as pro-inflammatory serum biomarkers in endometriosis. Using a decision tree-based supervised learning approach, we additionally present a straight-forward way of a potential clinical employment, aiming at less invasive (non-surgical) diagnosis.

**Supplementary Information:**

The online version contains supplementary material available at 10.1038/s41598-025-05719-1.

## Introduction

Several factors, including non-specific clinical symptoms, delays in diagnosis, and a general lack of non-invasive diagnostic techniques, not only highlight the current need for new scientific advancements in the field of endometriosis research, but also elucidate the widespread adoption of artificial intelligence, specifically machine- learning, within this domain. By training various mathematical algorithms with individually selected input parameters (e.g., general clinical parameters, biomarkers^1–4^metabolite/Raman spectra^5,6^genetic data^7,8^), a variety of clinically relevant problems can be addressed, ranging from prognosis prediction to the evaluation of non-invasive diagnostic methods. A recent review by Sivajohan et al. reported a pooled sensitivity of 81.7 to 96.7% for th use of machine-learning based analyses in the diagnosis and prediction endometriosis; albeit with regard to the underlying use-case (e.g., prediction of prognosis, prediction of outcome, or adjusting personalized treatment)^9^. Thus far, it has been suggested that endometriosis is linked to immune-inflammatory processes that occur in the pelvic peritoneal area and manifest as abnormal expression of hepcidin^10,11^soluble urokinase-type plasminogen activator receptor (suPar)^12^and/or interlukin-6 (IL-6)^13,14^. Since a broad range of studies from different research groups strongly support the use of the cytokine IL-6 as a potential marker regarding the diagnosis or pain intensity of endometriosis (and even endometriosis-associated infertility)^15,16^a comprehensive review indeed identified IL-6 as the most studied cytokine within endometriosis^13^. In contrast less is known about association of endometriosis with serum levels of hepcidin (an acute-phase protein involved in iron homeostasis – studies described higher levels of hepcidin within endometriosis^10^) or the protein suPar, which is activated during inflammatory processes^17,18^. While cell culture studies propose a pathophysiological role of suPar secretion within endometriosis patients, e.g., when analyzing endometrial biopsies, reliable studies evaluating its role as a serum marker remain scarce^12,19^. Summing up, the precise pathophysiological function of these proteins, whether as a concomitant phenomenon, markers of endometriosis, or potential triggering agent, remains to be resolved.

The objective of this study is to examine serum concentrations of hepcidin, suPar, and IL-6 as potential biomarkers for the non-invasive diagnosis of endometriosis, as well as their correlation with disease severity, as determined by the revised American Society for Reproductive Medicine (rASRM) score. Furthermore, our aim was to utilize a supervised learning methodology that relies on the aforementioned serum biomarkers in order to differentiate between individuals diagnosed with endometriosis and a non-endometriosis control group.

## Materials and methods

### Patient data

A total of 87 patients were included in the study, including 59 women with histomorphologically confirmed evidence of endometriosis (defined by the presence of at least two out of the three parameters: ectopic endometrioid glandular structures, CD10 + endometrial stromal cells, bleeding residuals with hemosiderin/hematoidin and macrophage appearance) and 28 women with no evidence of endometriosis (control group). Cases within the control group underwent gynecologic therapy due to alternative diseases (infertility, fibroids, other benign lesions of the uterus or ovaries). All study patient cases underwent treatment and surgery at the Department of Gynecology and Obstetrics at Saarland University between 2013 and 2019. Subsequently, a sequential histomorphological investigation was conducted at the Institute of Pathology at Saarland University. As exclusion criteria we a priori defined: “patients with recurrence/postmenopausal endometriosis”, “patients with systematic inflammatory processes”, “hematological diseases (e.g., anemia)”, “diseases of iron metabolism”, or “neoplastic diseases”. See Supp. Table 1 for a tabular summary of our inclusion and exclusion criteria. In line with standard practice, endometriosis surgery is conducted preferably during follicular phase. By convention, a previous hormonal therapy should be discontinued 8 weeks prior to surgery. Within our non-endometriosis control group, patients in this clinical trial were enrolled throughout their menstrual cycle, reflecting a certain degree of variability that occurs in daily clinical practice; controls did not receive hormonal therapy. All data were handled according to the Declaration of Helsinki and the study protocol was approved by the regional ethics committee (Ethics Committee of the Saarland Medical Association; approval no. 46/21). Therefore, all study methods were performed in accordance with the relevant national regulations.

### Serum samples and analysis of inflammatory serum parameters

All included cases’ serum samples were taken from our department’s endometriosis biobank, which comprises clinical patient data as well as serum samples and histopathological information from patients treated at our clinic. Prior to participation written informed consent was obtained from all individuals enrolled in this study; consecutive blood sample collection was performed at the day of surgery and stored in a deep freezer at -80 °C. For this study, serum levels of hepcidin (Human Hepcidin Quantikine ELISA kit; R&D Systems, USA), IL-6 (Human IL-6 Quantikine ELISA kit; R&D Systems, USA) and suPar (suPARnostic^®^ AUTO Flex ELISA kit; ViroGates A/S, Denmark) were tested in our laboratory employing Enzyme-linked Immunosorbent Assays (ELISA) kits according to the protocol established by the manufacturer. Additionally, serum levels of CRP, ferritin and soluble transferrin receptor (sTfR) were tested in the central laboratory of our hospital according to current clinical standards. Clinical parameters and laboratory data of the diagnostic routine (e.g., hemoglobin) were taken from the internal digital medical record.

### Statistical analysis and machine-learning

A preliminary assessment of normality was performed using the Shapiro-Wilk test. In order to examine potential correlations within the dataset, a Spearman-Rho analysis was conducted. Additionally, the Mann-Whitney test was used to assess variable differences between the endometriosis group and the control group, and the Kruskal-Wallis Test was employed to evaluate differences between three or more groups (rASRM stage). Hereby, the cut-off for statistical significance was set at *p* < 0.05. For a subsequent machine-learning analysis, the data was initially visualized and explored using non-linear t-distributed Stochastic Neighbor Embedding (t-SNE). For our supervised learning approach, the data were split into a training cohort (77 patients) as well as an external validation cohort (10 patients: 6 patients with endometriosis and 4 patients as controls); the latter serves useful to detect potential tendencies of overfitting within a trained model. Within this study an approximate time-based splitting approach was established, integrating endometriosis patients as well as controls last treated in our clinic into the external validation cohort. Classifier training and selection as well as hyperparameter optimization of the selected model was performed using the MATLAB Classification Learner App within the MATLAB Toolbox (MathWork, Natick, MA) for Statistics and Machine-Learning™. Hereby, endometriosis/no endometriosis were set as response parameters and hepcidin/suPar/IL-6 serum levels served as predictor variables. For internal classifier validation, the method of 5-fold cross validation was chosen. The minimum Redundancy – Maximum Relevance (MRMR) algorithm allowed for identification of feature importance. Classifier performance was assessed using standard metrics such as sensitivity, specificity, positive predictive value, negative predictive value, as well as AUROC curve. While the evaluation of serum inflammatory parameters follows a pre-planned study strategy, our consecutively employed machine learning analysis was performed as an exploratory (post-hoc) approach.

## Results

### Clinical characteristics of women with and without endometriosis

Women with endometriosis (refer to Supp. Table 2 for detailed listing of localization) as well as without endometriosis were matched in age [median (range) (years): 30 (19–40) vs. 29 (22–41) respectively] and with respect to body mass index (BMI) [median (Range) (kg/m^2^): 22.4 (17.6–37) vs. 22.1 (17.7–35.8) respectively]. Table 1 presents an overview of additional clinical variables, such as a menstrual cycle, catamenial pain, dysmenorrhea, dyspareunia, and hormonal contraception.

**Table 1:** Clinical characteristics of our endometriosis group as well as our control group.

	Control group (*n* = 28)	Endometriosis group (*N* = 59)
Age (years) [median (range)]	30 (19–40)	29 (22–41)
BMI (kg/m^2^) [median (range)]	22.4 (17.6–37)	22.1 (17.7–35.8)
Regular menstrual cycle		
Yes	21 (75%)	27 (46%)
No	7 (25%)	14 (24%)
Not known	0 (0%)	18 (30%)
Pain		
Yes	0 (0%)	36 (61%)
No	28 (100%)	19 (32%)
Not known	0 (0%)	4 (7%)
Dysmenorrhea		
Yes	0 (0%)	31 (52%)
No	28 (100%)	23 (39%)
Not known	0 (0%)	5 (9%)
Dyspareunia		
Yes	0 (0%)	12 (20%)
No	28 (100%)	42 (71%)
Not known	0 (0%)	5 (9%)
Hormonal contraception		
Yes	11 (39%)	19 (32%)
No	17 (61%)	37 (63%)
Not known	0 (0%)	3 (5%)

### Association of inflammatory biomarkers in endometriosis patients

Initial univariate analysis determined different levels of suPar (Mann–Whitney test; *p* = 0.024) and IL-6 (Mann–Whitney test; *p* < 0.001) between the endometriosis group and our control group; levels of hepcidin, CRP, sTfR and ferritin did not vary significantly between groups, refer to Table 2 for an optical display. With regard to the clinical patient characteristics age and BMI, a consecutive Spearman correlation analysis determined a distinct association of IL-6 (endometriosis group: *r* = 0.426, *p* < 0.001; control group: *r* = 0.724, *p* < 0.001) and patient’s BMI in both groups but, interestingly, a significant association of suPar concentration with both age (*r* = 0.462, *p* = 0.013) and BMI (*r* = 0.601, *p* < 0.001) in solely our non-endometriosis control group whereas not in patients with endometriosis (BMI: *r* = 0.165, *p* = 0.231; age: *r* = 0.032, *p* = 0.811), see also (Table 3). While hepcidin levels initially did not differ between our cohorts, a subsequent analysis of our groups’ percentile rankings (Q25, Q25-50, Q75) indicates differences in relative positions within the respective data sets collected [Q75 ranks: median (Range) (pg/ml): 26031 (17947–34973) in the endometriosis group (*n* = 15) vs. 14869 (12914–22755) in the control group (*n* = 7; Mann–Whitney test, *p* = 0.002)]. Therefore, all three inflammatory parameters (suPar, hepcidin, IL-6) collected in the studies were included in a further machine learning classification for the diagnosis of endometriosis. Evaluating our inflammatory markers of interest with regard to the stage of endometriosis (rASRM), we did not determine any association between the degree of inflammation and the clinical affection of endometriosis (Kruskal-Wallis Test; Hepcidin and rASRM I-IV: *p* = 0.492, suPar and rASRM I-IV: *p* = 0.100, IL-6 and rASRM I-IV: *p* = 0.626), see (Supp. Fig. 1).

**Table 2:** Results of serum level of inflammatory biomarker put to test, presented as median (range). p-value was determined using Mann-Whitney-test.

	Control group (*n* = 28)	Endometriosis group (*n* = 59)	*p*-value
Hepcidin (pg/ml)	8538 (153-22755)	9534 (127-34973)	NS
suPar (ng/ml)	1.9 (1.3–3.6)	2.3 (0.8–4.2)	0.024
IL-6 (pg/ml)	1.3 (0.4-6.0)	2.9 (0.3–50.3)	< 0.001
CRP (mg/ml)	1.2 (0.5–13.7)	1.1 (0.5–26.6)	NS
sTfR (mg/ml)	2.5 (1.6–4.9)	2.6 (1.9–7.2)	NS
Ferritin (ng/ml)	40.5 (8-118)	55.0 (6-194)	NS

**Table 3:** Correlation analysis of SuPar and IL-6 with clinical parameters according to each a priori defined subgroup (endometriosis/control).

	Control group		Endometriosis group	
	Age	BMI	Age	BMI
suPar ng/ml	*r* = 0.462 *p* = 0.013	*r* = 0.601 *p* < 0.001	*r* = 0.032 *p* = 0.811	*r* = 0.165 *p* = 0.213
IL-6 pg/ml	*r* = 0.042 *p* = 0.883	*r* = 0.724 *p* < 0.001	*r* = 0.124 *p* = 0.351	*r* = 0.426 *p* < 0.001

### Machine learning assisted diagnosis of endometriosis based on inflammatory biomarkers

**Fig. 1:**
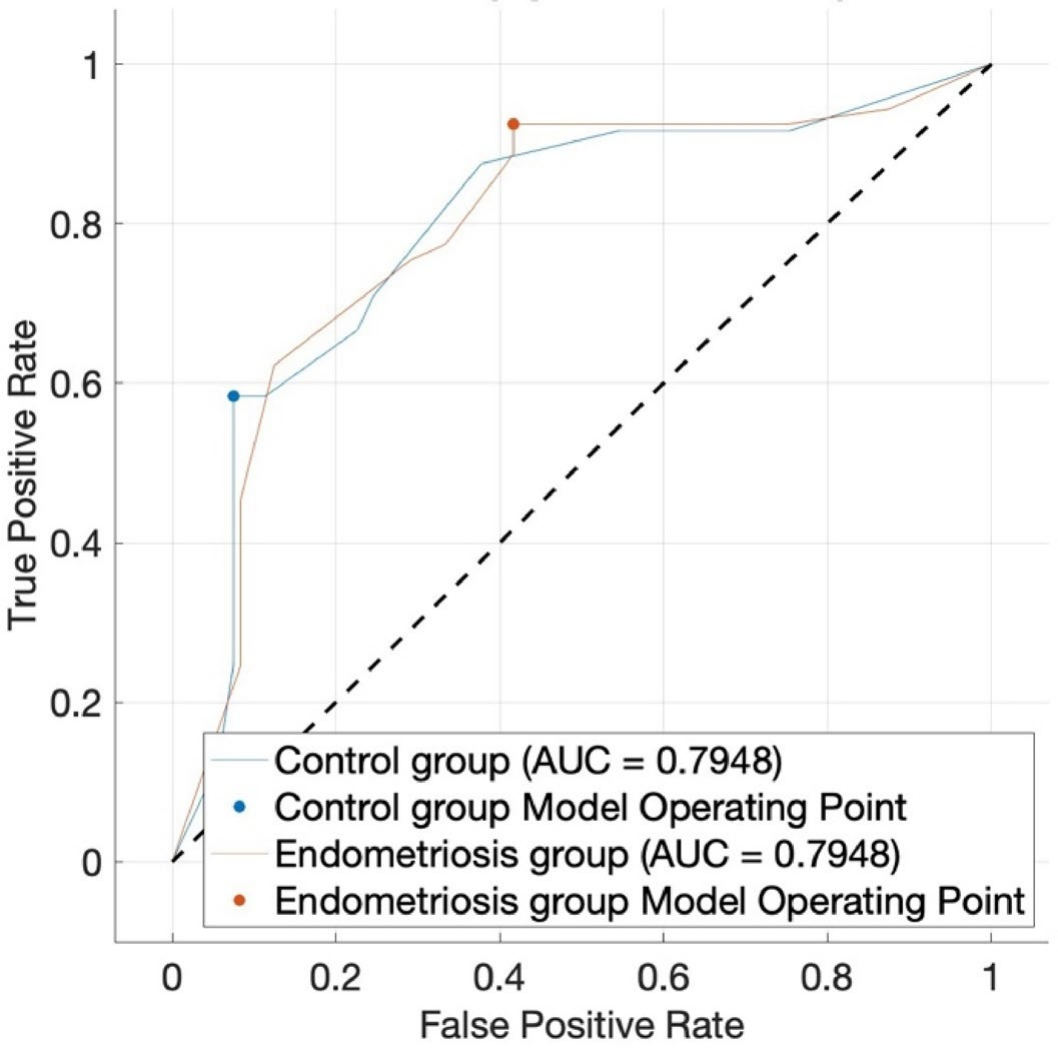
displays the ROC curve of our supervised learning approach (decision tree) for the classification of endometriosis based on serum levels of suPar, hepcidin, IL-6. The dotted line represents a random-chance classification.

**Fig. 2:**
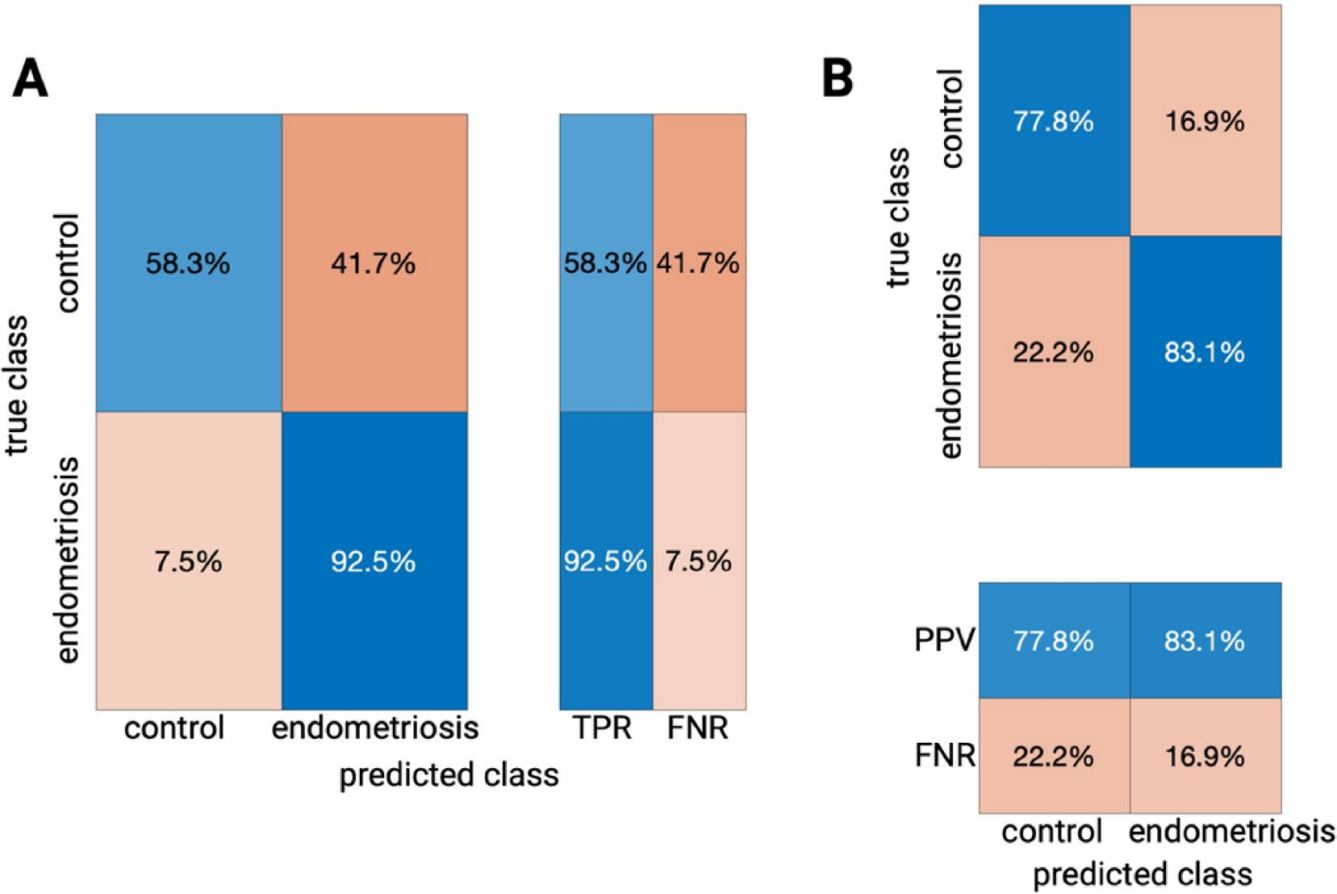
Confusion matrices depicting both PPV (positive predictive value)/FDR (false discovery rate) as well as TPR (true positive rate)/FNR (false negative rate) of our decision tree classification and our training data set based on the internal 5-fold cross validation.

**Fig. 3:**
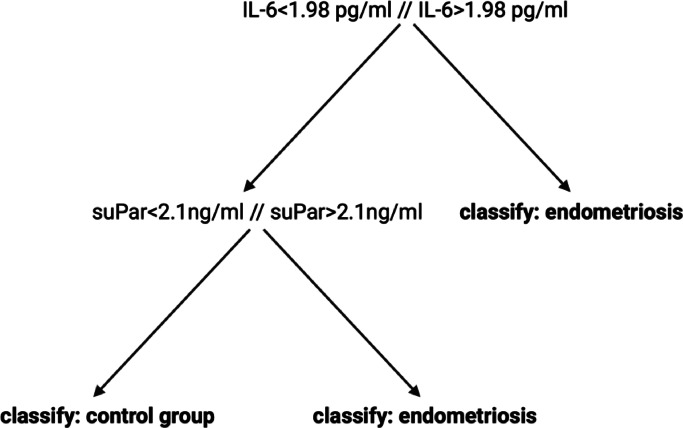
Individual cut-off points of our decision tree classifier displayed from the top node including all leaf nodes and branches. *suPar* soluble urokinase-type plasminogen activator receptor, *IL-6* interleukin-6.

A preliminary data display (t-SNE) did not reveal any relevant similarities between our groups. To establish a profound algorithm performance, we split our data into a training cohort for classifier training and an external validation cohort for following testing of the algorithm’s performance accuracy on new data points as mentioned above. Using a training set of 77 patients (53 of the endometriosis group, 24 of the control group) and an internal 5-fold cross validation we employed a decision tree classifier which resulted in an overall accuracy (right classifications/all classifications) of 81.8% with corresponding AUROC value = 0.7948. Figure 1 shows the ROC curve, here for both classes defined (default option when using the MATLAB Classification Learner App). Hereby, the TPR (true positive rate) for endometriosis detection was 92.5% and the TPR for our control group was 58.3%, the resulting PPV (positive predictive value) for diagnosing endometriosis based on our established machine learning algorithm and the aforementioned inflammatory serum biomarkers put to test was 83.1%, the PPV for excluding endometriosis in a patient not affected by the disease was 77.8%; see (Fig. 2). As suggested by our preceding analysis, determination of feature importance proved IL-6 (MRMR, importance score: 0.0897) and suPar (MRMR, importance score: 0.0246) as most useful for diagnostic purposes. Figure 3 additionally depicts internal cut-off points of our decision tree classifier that can manually be followed from the top node to leaf nodes following the branches in dependence of the predictor’s value. When testing our established classifier on the holdout external validation set (6 of the endometriosis group, 4 of the control group), we confirmed our reported metrics with an overall accuracy of 80.0%, suggesting solid classification capabilities without any suspicious signs of potential overfitting.

## Discussion

Reciprocal relationships between existing endometriosis and increased inflammatory parameters have been confirmed in several studies. From a pathophysiological point of view interactions between ectopic endometrial tissue and pathways of the innate and adaptive immune system as well as regional inflammatory processes have been described so far and are reflected in altered immune cell function but also impaired peritoneal immunosurveillance^20,21^. However to date, it is not been fully understood whether such phenomena are causative conditions in the etiopathogenesis of endometriosis or vice versa represent reactive conditions^22^.

These findings, which are originally often based on translational research results^23–25^are also reflected by numerous diagnostic studies focusing for example on the pro- inflammatory cytokine IL-6 in patients with endometriosis; IL-6 is produced by macrophages, which are among the most prevalent cell types within endometriomas and are frequently observed in close proximity to peritoneal ectopic endometrial lesions. In that regard the team of Kokot et al. determined elevated IL-6 serum levels in patients with endometriosis in comparison to a healthy control group and furthermore described a stage dependent increase in concentration (rASRM Stage III vs. Stage IV; *p* = 0.0409)^26^. Taking a different approach, the team of Ghodsi et al. analyzed IL-6 concentrations within follicular fluids from endometriosis patients and determined a higher concentration in comparison to healthy controls^27^and Incognito et al. examined in a systematic approach the relevance of IL-6 as predictive marker for endometriosis-associated infertility^13^. However, an ultimate step of transformation of these findings from benchmark to clinical routine has not yet been fulfilled. It is precisely this gap that some research teams have been addressing recently: by using machine-learning algorithms, they are proposing an alternative way of analyzing data to promote the development of biomarker-based diagnostics towards clinical utility. Vodolazkaia et al. employed inter alia a least squares support vector machines based multivariate analysis of selected plasma biomarkers (VEGF, annexin V, CA-125, glycodelin) in order to detect endometriosis even in patients without prior sonographical evidence (training data set: accuracy 81%; test data set: accuracy 74%)^1^. In contrast to this, Knific et al. analyzed 40 cytokine plasma biomarkers in 116 endometriosis patients and a healthy control group and did not determine acceptable accuracies when using different machine-learning algorithms (e.g., random forest analysis, decision tree) for classification^4^.

Within our study, we report the machine-learning based classification of endometriosis based on inflammatory serum biomarkers with an internal overall accuracy of 81.8%; our proposed model performs sufficiently comparable even when classifying new data (external validation data set). As revealed already by MRMR algorithm analysis, a closer look at the decision-making basis of our decision tree proves the importance of IL-6 (> 1.98 pg/ml) and suPar (> 2.1 ng/ml) within our supervised learning approach – both markers have been shown to be significantly altered within the endometriosis group even in our preceding univariate analysis. Therefore, our findings are in line with current literature proposing a significant role of suPar within the inflammatory process in patients with endometriosis^28^.

From a critical perspective, the overall sample size of 87 patients hampered the additional uptake of further predictor variables - such as clinical symptoms – which would potentially allow for an improved classification ability of the established algorithm; however, we refrained from doing so in order to ensure a proper relationship between sample size and number of predictor variables (*one in ten rule*)^29^. That said, it is worth mentioning that even in the small field of endometriosis research different types of predictor parameters are put to test, aiming for classification/diagnosis relying on clinical symptoms or imaging variables^9^. Prospectively, studies with a sufficient number of participants could take into account a larger combination of most promising predictor variables. Additionally, future multi-center study approaches would allow for algorithm validation with further diversified test data sets. Within the scope of this study, solely a selected panel of parameters of interest could be analyzed, leaving out traditional markers in the field of gynecological research such as CA125 (MUC-16, mucin-16), estrogen, or anti-müllerian hormone (AMH). While a study of Somigliana et al. in 2004 did not postulate any significant diagnostic advantages of testing both IL-6 and CA125, future study approaches may not only determine the association of suPar as well as Hepcidin with e.g., CA125, but prospectively also their combined prognostic potential^30^. Last but not least, potential confounders possibly arising from individual constitutional differences, inter-individual hormonal discrepancies, or non-controlled environmental influences (e.g., smoking) could always hamper the presented study results. Albeit we thoroughly controlled for known potential confounders, which may impact individual systematic inflammatory processes (refer to Supp. Table 1), a potential risk of (yet) unknown bias always needs to be considered when cautiously interpreting data from a clinical study.

## Conclusion

To sum up: The results presented in our study highlight the diagnostic potential not only of IL-6 but recognizable also suPar as a pro-inflammatory serum biomarker in endometriosis patients. By establishing a supervised machine learning algorithm that provides robust accuracy even on an external validation dataset, we introduce a straightforward computational method for integrating our findings into a concrete clinical tool, opening up future prospects for non-surgical diagnostic methods.

## Electronic supplementary material

Below is the link to the electronic supplementary material.

Supplementary Material 1

## Data Availability

Interested parties are warmly invited to contact the corresponding author for individual options.
