# Supplementary material for: Evaluation of inflammatory serum parameters as a diagnostic tool in patients with endometriosis: a case-control study
Source: Sci Rep. 2025 Jun 20;15:20172. doi: 10.1038/s41598-025-05719-1 (PMC12181322; doi:10.1038/s41598-025-05719-1)
Supplement: Supplementary file 1 — Supplementary Material 1 [file 41598_2025_5719_MOESM1_ESM.docx]

**SUPPLEMENTARY MATERIAL**

**Title:** Evaluation of Inflammatory Serum Parameters as a Diagnostic Tool in Patients with Endometriosis: a case-control study

**Supp. Table 1**. Endometriosis study cohort inclusion and exclusion criteria summary.

| **inclusion criteria** | **exclusion criteria** |
| --- | --- |
| histomorphologically diagnosed endometriosis | recurrence of endometriosis or postmenopausal endometriosis |
| patient gave informed consent for study participation and biobank inclusion | patients with the following pre-existing conditions: systematic inflammatory processes, hematological diseases, affections of iron metabolism, neoplasias |

**Supp. Table 2**. Depiction of the localization of the disease, according to each individual participant.

| **no of patient** | **localization** |
| --- | --- |
| 1 | right pelvic wall, bladder peritoneum |
| 2 | scar endometriosis |
| 3 | left pelvic wall, douglas space |
| 4 | both sides pelvic wall, douglas space |
| 5 | both sides pelvic wall, abdominal wall, douglas space |
| 6 | abdominal and/or pelvic wall |
| 7 | both sides pelvic wall, bladder peritoneum |
| 8 | left ovary |
| 9 | douglas space |
| 10 | right abdominal wall, bladder peritoneum, right pelvic wall left ovary, vagina, rectum |
| 11 | scar endometriosis |
| 12 | bladder peritoneum |
| 13 | bladder peritoneum, left ovary, right pelvic wall |
| 14 | right ovary, left pelvic wall |
| 15 | douglas space |
| 16 | abdominal wall, pelvic wall |
| 17 | douglas space and bladder peritoneum |
| 18 | douglas space, both ovaries |
| 19 | right pelvic wall, rectum |
| 20 | douglas space, right pelvic wall, left ovary, bladder peritoneum |
| 21 | vagina, right pelvic wall, rectum |
| 22 | right pelvic wall |
| 23 | left pelvic wall, bladder peritoneum |
| 24 | left pelvic wall, rectum, right pelvic wall, bladder peritoneum, both ovaries |
| 25 | pelvic wall both sides |
| 26 | left ovary, left pelvic wall, vagina |
| 27 | abdominal wall |
| 28 | ovary both sides, vagina |
| 29 | bladder peritoneum |
| 30 | left ovary |
| 31 | scar endometriosis |
| 32 | right pelvic wall, rectum |
| 33 | left pelvic wall, douglas space |
| 34 | right pelvic wall, bladder peritoneum |
| 35 | left pelvic wall |
| 36 | douglas space, colon |
| 37 | bladder peritoneum, douglas space |
| 38 | douglas space, right ovary, adenomyosis |
| 39 | ovary |
| 40 | pelvic wall, rectum |
| 41 | left ovary, douglas space, right pelvic wall |
| 42 | left ovary |
| 43 | left ovary |
| 44 | left ovary |
| 45 | left pelvic wall |
| 46 | right pelvic wall, rectum |
| 47 | left pelvic wall |
| 48 | douglas space |
| 49 | right ovary, pelvic wall |
| 50 | douglas space |
| 51 | douglas space, right pelvic wall |
| 52 | pelvic wall, rectum |
| 53 | douglas space, pelvic wall both sides |
| 54 | douglas space, pelvic wall left |
| 55 | ovary both sides |
| 56 | left pelvic wall |
| 57 | left pelvic wall |
| 58 | douglas space |
| 59 | right pelvic wall |


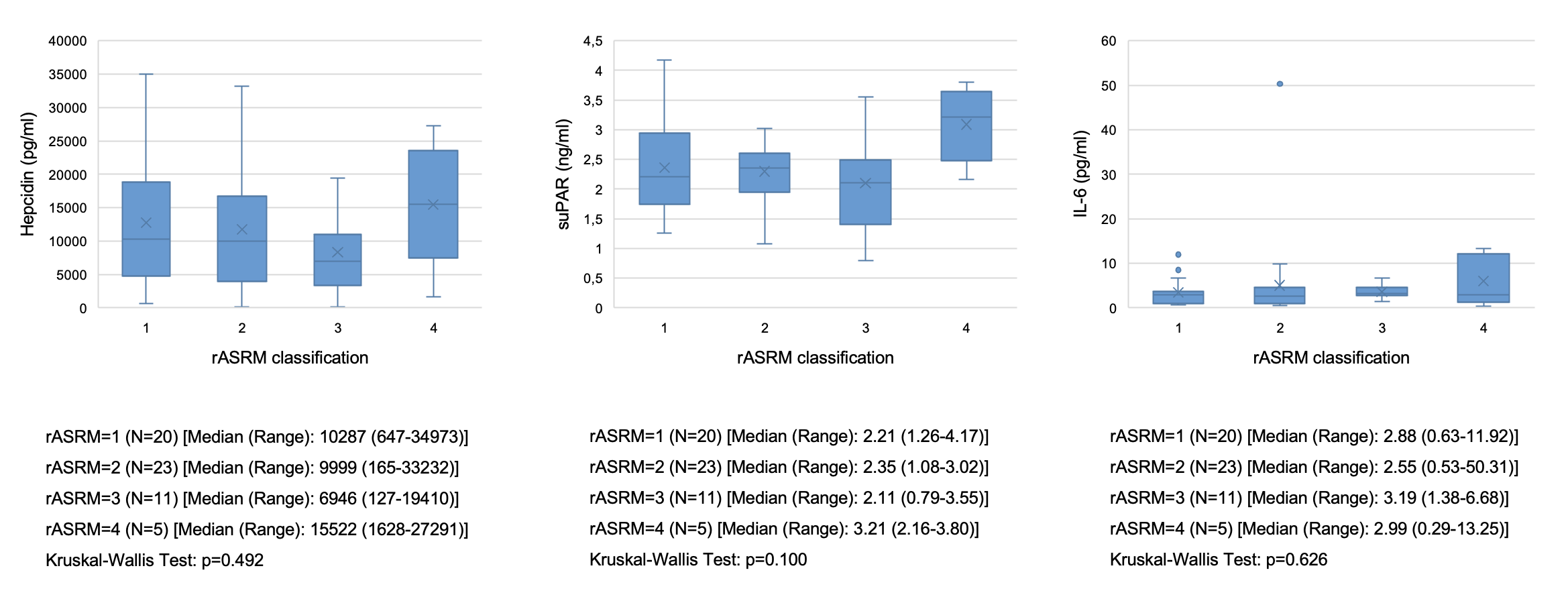


**Supp. Figure 1**. Inflammatory markers of interest with regard to the stage of endometriosis (rASRM).
